# A laboratory simulation of *Arabidopsis* seed dormancy cycling provides new insight into its regulation by clock genes and the dormancy‐related genes *DOG1*, *MFT*, *CIPK23* and *PHYA*

**DOI:** 10.1111/pce.12940

**Published:** 2017-05-16

**Authors:** Steven Footitt, Hülya Ölçer‐Footitt, Angela J. Hambidge, William E. Finch‐Savage

**Affiliations:** ^1^ School of Life Sciences, Wellesbourne Campus University of Warwick Warwick Warwickshire CV35 9EF UK; ^2^ Department of Biology, Faculty of Arts and Sciences, Evliya Celebi Campus Dumlupınar University TR‐43100 Kütahya Turkey

**Keywords:** circadian clock, circannual rhythm, germination, thermal time

## Abstract

Environmental signals drive seed dormancy cycling in the soil to synchronize germination with the optimal time of year, a process essential for species' fitness and survival. Previous correlation of transcription profiles in exhumed seeds with annual environmental signals revealed the coordination of dormancy‐regulating mechanisms with the soil environment. Here, we developed a rapid and robust laboratory dormancy cycling simulation. The utility of this simulation was tested in two ways: firstly, using mutants in known dormancy‐related genes [DELAY OF GERMINATION 1 (*DOG1*), MOTHER OF FLOWERING TIME (*MFT*), CBL‐INTERACTING PROTEIN KINASE 23 (*CIPK23*) and PHYTOCHROME A (*PHYA*)] and secondly, using further mutants, we test the hypothesis that components of the circadian clock are involved in coordination of the annual seed dormancy cycle. The rate of dormancy induction and relief differed in all lines tested. In the mutants, *dog1‐2* and *mft2*, dormancy induction was reduced but not absent. *DOG1* is not absolutely required for dormancy. In *cipk23* and *phyA* dormancy, induction was accelerated. Involvement of the clock in dormancy cycling was clear when mutants in the morning and evening loops of the clock were compared. Dormancy induction was faster when the morning loop was compromised and delayed when the evening loop was compromised.

## Abstract 2

We present a robust laboratory protocol for rapid seed dormancy cycling in *Arabidopsis*. Using a range of mutants in a circadian clock and dormancy‐related genes, we demonstrate a role for the clock in dormancy cycling and the induction of dormancy in the *DOG1* mutant. This presents a new tool for the genetic dissection of dormancy cycling and comparisons of cycling behaviour across ecotypes.

## Introduction

Seeds are highly efficient sensors and interpreters of the prevailing environment and their environmental history. Seeds first sense the maternal environment to set the depth of primary dormancy at maturity (e.g. temperature) (Kendall *et al.* 2011; Penfield and Springthorpe 2012; He *et al.* 2014; Huang *et al.* 2014, 2015; Chen *et al*. 2014). Seeds that do not germinate immediately upon shedding enter the soil seed bank and respond to the soil environment by continually adjusting the depth of dormancy to time the eventual completion of germination (Footitt *et al.* 2011, 2013, 2014, 2015; Finch‐Savage and Footitt 2012, 2017; Penfield and Springthorpe 2012). When depth of dormancy is low, seeds are sensitive to signals that inform of the spatial environment (e.g. light, nitrate and temperature). If these signals are not received to remove the final layer of dormancy, then seeds enter secondary dormancy (Finch‐Savage and Footitt 2017). In this way, seeds determine the time and place of plant establishment to synchronize their life cycle with favourable environments (Finch‐Savage and Leubner‐Metzger 2006; Springthorpe and Penfield 2015; Burghardt *et al.* 2016). Recent correlations of annual gene expression patterns in exhumed seeds with environmental signals in the field provided the first insight into the temporal integration of the molecular regulation of dormancy cycling (Footitt *et al.* 2011, 2013, 2014).

However, studying dormancy cycling in the field is a long‐term undertaking, and ethical and regulatory reasons can preclude the use of seeds from genetically modified plants to dissect the role of individual genes; progress in understanding has therefore been slow. To address this in the work presented, we used our field and laboratory observations (Cadman *et al.* 2006; Footitt *et al.* 2011; Huang *et al.* 2015) to develop a representative, but rapid and simple laboratory simulation of dormancy cycling. It enables a full dormancy cycle to be completed in approximately 8 weeks. The method was developed using the deeply dormant winter annual ecotype Cape Verde Island (Cvi) and then applied to the Col‐0 and Ler ecotypes to facilitate mutant analyses.

Here, we explore the utility of this laboratory simulation in two ways.

Firstly, we use the simulation to confirm the involvement of genes previously identified in correlative gene expression studies as central to the regulation of seed dormancy cycling (Footitt *et al*. 2011, 2013). These studies suggest that by influencing the central integrating hormonal balance [abscisic acid (ABA)/gibberellins (GA)], DELAY OF GERMINATION 1 (*DOG1*) and MOTHER OF FLOWERING TIME (*MFT*) play key roles in the response to temporal signals (e.g. temperature) that regulate dormancy cycling (Finch‐Savage and Footitt 2017). This is linked to temporal changes in the expression of PHYTOCHROME A (*PHYA*) and CBL‐INTERACTING PROTEIN KINASE 23 (*CIPK23*) (CBL, CALCINEURIN B_LIKE PROTEIN) that alter sensitivity to signals indicating suitability for germination completion (spatial signals: light and nitrate, respectively). Once sensitized, seeds respond to these signals through the ABA/GA balance to bring about the completion of germination when conditions are optimal (Finch‐Savage and Footitt 2017). We confirm the involvement of these genes in ABA sensitivity and dormancy cycling using mutant lines (*dog1‐2*, *mft2*, *cipk23* and *phyA*). In the field, dormancy induction and relief during cycling were shown to progress in thermal time (Footitt *et al*. 2011), and we use this approach to analyse data in the present work. Thermal time is quantified as the amount by which temperature exceeds a minimum temperature or threshold for the process in question. When this value is summed over days to give degree days (°C days), thermal time can be used to measure progress towards the completion of that process (Finch‐Savage and Leubner‐Metzger 2006).

Secondly, we use the simulation to test the previously unstudied hypothesis that components of the circadian clock may be involved in coordination of the annual seed dormancy cycle. The regulation of daily circadian rhythms has been extensively studied in plants (e.g. Salome and McClung 2005; Seung *et al*. 2012; Seo and Mas 2015; Atamian and Harmer 2016). In a 24 h cycle, interlocking morning and evening feedback loops control the period and phases of the circadian clock. The morning loop in *Arabidopsis* contains the MYB‐related transcription factors *LATE ELONGATED HYPOCOTYL* (*LHY*) and *CIRCADIAN CLOCK ASSOCIATED 1* (*CCA1*) whose increased expression represses the evening loop gene *TIMING OF CAB EXPRESSION* (*TOC1*). *LHY*/*CCA1* also induce the sequential expression of the *PSEUDO RESPONSE REGULATOR* (*PRR*) genes *PRR9*, *PRR7* and *PRR5*, which feedback during the day to progressively repress *LHY/CCA1* and therefore relieve repression of *TOC1*. The protein of the latter which induces *LHY/CCA1* expression (as reviewed in Hsu and Harmer 2014), is targeted for degradation by GIGANTEA (*GI*) in conjunction with ZEITLUPE (ZTL). *GI* then appears to be repressed by EARLY FLOWERING 3 (ELF*3*) a member of the evening complex (Mishra and Panigrahi 2015). The evening complex is formed by the proteins ELF3, ELF4 and LUX ARRYTHMO (LUX), and this represses the expression of the day‐phased clock gene *PRR9* (Hsu and Harmer 2014).

Some of these genes are known to influence seed dormancy. For example, *LHY* and *CCA1* mutants were more sensitive to dormancy relieving low temperature stratification and the *GI* mutant less sensitive (Penfield and Hall 2009). They also alter the hormone balance in seeds. For example, *GI* and *TOC1* mutants influence ABA and GA sensitivity and the expression of ABA‐related and GA‐related genes (Penfield and Hall 2009). The clock was also a key regulator of physiological activity when dormancy of imbibed Euphorbia esula seeds was relieved by alternating temperature in the dark (Foley *et al.* 2010). This indicates that the clock could respond to temperature signals in the dark conditions experienced in the soil seedbank; furthermore, temperature alternations of 4 °C are known to entrain the clock (Salome and McClung 2005). However, in seeds, it is the rhythm of the seasonal temperature cycle that provides a temporal signal indicating time of year (Probert 2000). In other tissues, components of the circadian clock can respond to seasonal changes in day length and associated alternation of light and temperature to coordinate tree bud dormancy with the time of year (Cooke *et al.* 2012). A similar role for the clock in seeds is largely unexplored. To address this, we use a targeted selection of mutant lines of clock genes to determine their contribution to the dormancy cycle and associated ABA sensitivity. We complement this by analysing the annual transcription profiles of these genes during dormancy cycling in the field.

## Materials and Methods

### Seed production

Seeds of the *Arabidopsis* Cvi and Burren (Bur) ecotypes were produced in a heated glasshouse with supplemental lighting in 2007 (Cvi) and 2008 (Bur) and were harvested, processed and then stored at −80 °C as described elsewhere (Footitt *et al*. 2011, 2013). Seeds of the *Arabidopsis* mutants' *toc1‐101* (Kikis *et al.* 2005), *lhy20 cca1‐1* (Yakir *et al.* 2009), *lhy20 cca1‐1 toc1‐2* (Yamashino *et al.* 2008), *prr5‐11 prr7‐11 prr9‐10* (Nakamichi *et al*. 2009), *dog1‐2* (Nakabayashi *et al.* 2012), *mft2* (Xi *et al.* 2010), *cipk23* (N503652) and *phyA* (N6223) are in the Col‐0 (N1092) genetic background, while the overexpressing lines *LHY‐OX* and *CCA1‐OX* are in the Ler and Col‐0 backgrounds, respectively (Green *et al.* 2002). All lines and their wild types were produced in the same growth cabinet (16 °/16 °C, 16 h L/8 h D). Following harvesting and processing seeds were stored at −80 °C (see Supporting Information methods for seed production conditions).

### Dormancy cycling in the laboratory

The annual variation in soil temperature and water potential are seen to impact the annual seed dormancy cycle in the field (Footitt *et al.* 2011). These observations were used to develop a protocol for dormancy cycling in the laboratory. Dormancy/germination experimental treatments and procedures used surface‐sterilized seeds and were all carried out in the dark under a green safe light unless otherwise stated.

### Impact of water potential on dormancy status in Cape Verde Island

Decreasing soil water potential was associated with low temperature induction of dormancy in Cvi in the field (Footitt *et al*. 2011). Consequently, its role was tested in the laboratory. Dormant seeds were surface sterilized in 2.5% dilution of domestic bleach for 5 min and washed three times in water. Seeds were then placed (3 × 40 seeds) into boxes (124 × 88 × 22 mm) (Stewart Plastics Ltd, UK). Each box contained 25 mL of solution set at a range of water potentials (0, −0.4, −0.8 and 1.2 MPa) using polyethylene glycol (PEG) 8000. This PEG solution volume represents a solution volume/paper weight ratio of 3.55 that minimizes the concentrating effect of filter paper on the solution (Hardegree and Emmerich 1990). This liquid reservoir was accommodated beneath the seeds as follows. In the base of each box was placed a piece of glass‐drying mat (Nisbits Ltd, UK). The drying mat was an open lattice 3 mm deep to create space for the PEG solution. On top of this was placed nylon mesh (1 mm mesh size) (Plastok, UK) to support the single sheet of Whatman 3MM chromatography paper (Camlab, UK) that is then placed on top. Strips of nylon mesh (125 *μ*m mesh size, 45% open mesh) (Plastok, UK) were then laid on the paper, and each seed replicate was placed on one of those individual strips (for a visual representation, see Fig. S1). Boxes were then sealed inside the freezer bags to minimize evaporation and wrapped in two layers of aluminium foil to exclude light and incubated at 5 °C for up to 14 d. Germination tests were carried out on these seeds after increasing intervals at 5 °C. The nylon strips holding the seeds were transferred to new boxes containing two sheets of chromatography paper and 8 mL of 50 or 250 *μ* m Gibberellin_4 + 7_ in citrate/phosphate buffer (pH 5.0) or a buffer control in the light at 20 °C and germination recorded over 28 d (Footitt *et al.* 2011). Gibberellin_4 + 7_ was dissolved in 100 *μ*L 0.1 m KOH before preparing the stock solution.

Seeds incubated on water (0 MPa) were also transferred at intervals to fresh water, or 10 mm KNO_3_ and incubated at 20 °C/light for 28 d to record germination. In all treatments, dark germinated seeds were recorded on transfer to the light. Germination was recorded as protrusion of the radicle through the seed coat and micropylar endosperm.

### Dormancy cycling in Cape Verde Island

The constrasting impact of winter and summer temperature on the annual dormancy cycle of Cvi was simulated using lower and higher constant temperatures to simulate dormancy cycling in the laboratory. Dormant seeds were plated (3 × 40 seeds) onto nylon mesh strips in boxes containing a −1.2 MPa PEG 8000 solution as mentioned previously and incubated at 5 °C for up to 21 d. At this point, seeds were transferred to boxes containing two sheets of chromatography paper and 8 mL water and incubated at 25 °C for 35 d. At each transfer point, dark germinated seeds were counted. At intervals, boxes were removed and dormancy tested by transferring seeds to boxes in the light containing 50 or 250 *μ* m Gibberellin_4 + 7_ or a buffer control as mentioned previously.

### Dormancy cycling in Col‐0 wild type and mutants

Using the laboratory dormancy cycling simulation, mutants in the Col‐0 genetic background were used to test the contribution of selected genes to dormancy cycling. Seeds were plated (3 × 40 seeds) into boxes containing a −1.0 MPa PEG 8000 solution as mentioned previously. A series of experiments testing a wide range of treatment temperatures and durations were then performed to evaluate the role of temperature in the induction and relief of dormancy. For the impact of cold conditioning on high‐temperature dormancy induction, seeds were incubated at 5 °C/−1 MPa for up to 28 d and then transferred to germination plates containing two sheets of chromatography paper and 8 mL of water and incubated at 20 (clock mutants only), 25 and 30 °C for up to 14 d at which point plates were transferred to 5 °C for up to 29 d. At intervals during each incubation period, boxes were removed for germination testing at 25 °C/light for 14 d. Loss of dormancy in the presence of nitrate in Col‐0 and *cipk23* seeds subjected to 5 °C/−1 MPa followed by 25 °C was also tested by transferring seeds to 10 mm KNO_3_ at 25 °C/light for 14 d. Germination tests were carried out at 25 °C as the Col‐0 wild type retains greater thermodormancy at this temperature compared with 20 °C. Seeds on PEG 8000 solution were transferred to water prior to germination testing in the light. Dark germination was recorded at each transfer point and prior to germination testing.

### Abscisic acid sensitivity

As changing ABA sensitivity has in integral role in the dormancy continuum, the sensitivity of mutants was determined. Seeds were plated onto nylon mesh in boxes containing water as mentioned previously. They were cold stratified at 5 °C/dark for 3 d and then transferred to boxes containing 10–250 nm (±)‐ABA (Sigma, UK) in citrate/phosphate buffer (pH 5.0) and incubated in the light at 25 °C. ABA was dissolved in 100 *μ*L 0.1 m KOH before preparing the stock solution.

### Dormancy cycling in the field and gene expression analysis

As seed dormancy cycling displays an annual rhythm in response to seasonal soil temperature patterns, we determined the transcriptional profile of seven clock genes in seeds recovered over 12 months from field soil. Experiments on dormancy cycling in the field were performed as described previously (Footitt *et al.* 2011, 2013). Seeds were recovered from the soil in the morning of the day of harvest. Quantitative PCR (QPCR) of circadian clock gene expression was performed using the touchdown PCR thermal cycle: one cycle at 95 °C for 10 min followed by 50 cycles at 95 °C for 30 s, 70 °C (decreasing by 0.2 °C/cycle to a target temperature of 67 °C) for 30 s and 72 °C for 30 s. All other details regarding QPCR procedures and analysis were described previously (Footitt *et al.* 2015). Primer sequences are given in Table S2.

## Results

### Dormancy cycling in Cape Verde Island under laboratory conditions

**Figure 1:**
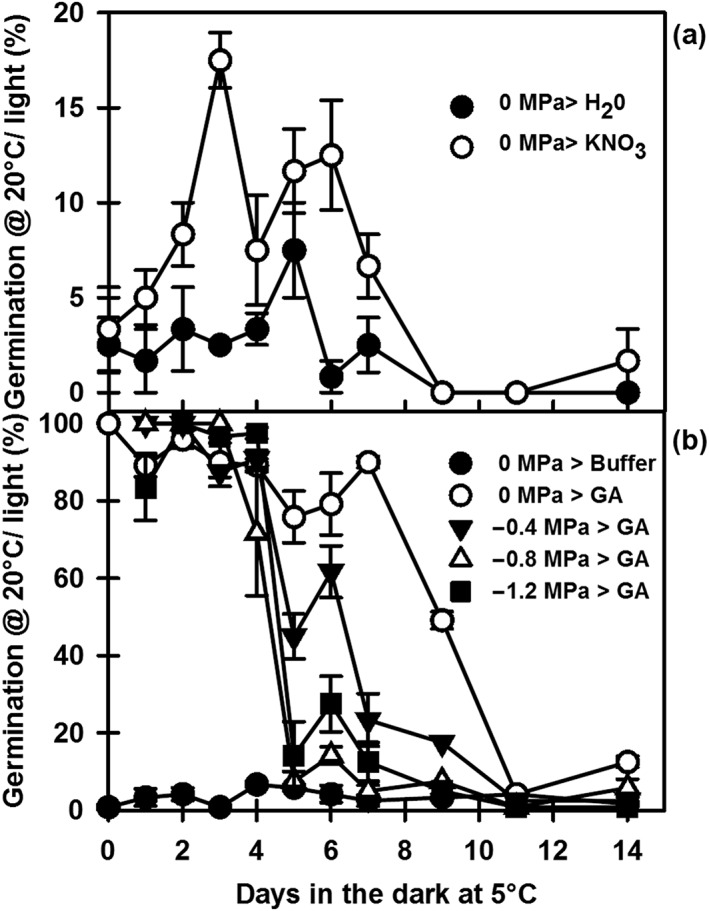
Induction of secondary dormancy in Cape Verde Island (Cvi) in response to cold stratification and decreasing water potential. Primary dormant Cvi seeds were incubated at 5 °C/dark on water or a range of water potentials from −0.4 to −1.2 MPa. At increasing periods of time, dormancy status was determined by measuring germination following transfer of seeds to (a) water or 10 mm KNO_3_ or (b) a buffer control or 250 *μ* m GA_4 + 7_ buffered at pH 5.0 at 20 °C/light for 28 d. Data are mean ± SE (*n* = 3). Absence of error bars indicates that SE is smaller than the symbol. GA, gibberellins.

Cape Verde Island seeds in field soil are induced into deeper primary dormancy by low temperatures in winter, and dormancy then declines to low levels in response to higher temperatures in spring/summer; deeper dormancy (secondary dormancy) is then reinduced by autumn/winter low temperatures (Footitt *et al*. 2011). A series of experiments were conducted to reproduce this behaviour in the laboratory. Primary dormant seeds on water did not germinate in the dark, and germination was less than 5% at 20 °C/light (Fig. 1a). Periods of pre‐exposure to low temperature in the dark up to 14 d had a limited effect on germination on water (Fig. 1a) and the buffer control (Fig. 1b). Germination on nitrate marginally increased from 5 to 17% in the first 4 d and then declined on further exposure. However, full germination was induced by imbibition on GA (250 *μ* m GA_4 + 7_), which then progressively declined on exposure to low temperature indicating an increasing depth of dormancy not evident on water, or the buffer control (Fig. 1b). Depth of dormancy increased more rapidly on exposure to low temperature when seeds were incubated in negative water potentials (down to −1.2 MPa) consistent with the observations of Auge *et al.* (2015).

**Figure 2:**
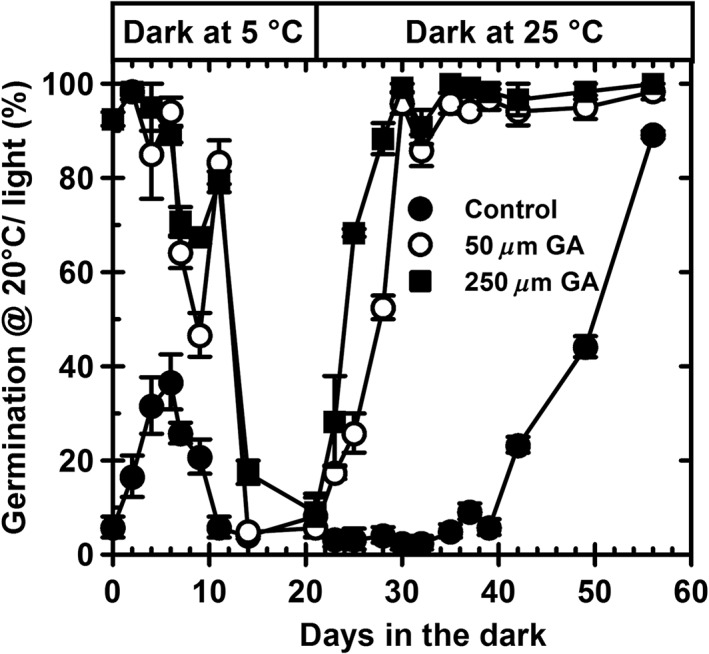
Simulated dormancy cycling in Cape Verde Island. Seeds were incubated at 5 °C/dark at −1.2 MPa for up to 21 d before being transferred to water at 25 °C/dark. At increasing periods of time, dormancy status was determined by measuring germination following transfer of seeds to a buffer control, 50 or 250 *μ* m GA_4 + 7_ buffered at pH 5.0 at 20 °C/light for 28 d. Data are mean ± SE (*n* = 3). Absence of error bars indicates that SE is smaller than the symbol. GA, gibberellins.

A second lot of seeds from the same harvest were exposed to low temperature (5 °C/dark) for 21 d with and without water stress at −1.2 MPa and then transferred to water at 25 °C/dark to simulate a full dormancy cycle (Fig. 2). This second seed lot had been stored at −20 °C, which resulted in a lower dormancy level. With these seeds, germination on the buffer control increased to <40% after 6 d of low temperature indicating that this proportion of the population had the lowest level of primary dormancy. In this portion, dormancy could then be relieved by light, with the remainder not yet light sensitive. With continued low‐temperature exposure, deeper dormancy was induced in the population as a whole. Sensitivity to GA_4 + 7_ declined (i.e. dormancy deepened) so that no seeds germinated even at 250 *μ* m GA_4 + 7_ after exposure to low temperature for 21 d. Depth of dormancy then declined progressively in the subsequent high‐temperature phase of the cycle. This began after 2 d on GA and then later in the control after 40 d.

### Dormancy cycling in Col‐0 and Ler

**Figure 3:**
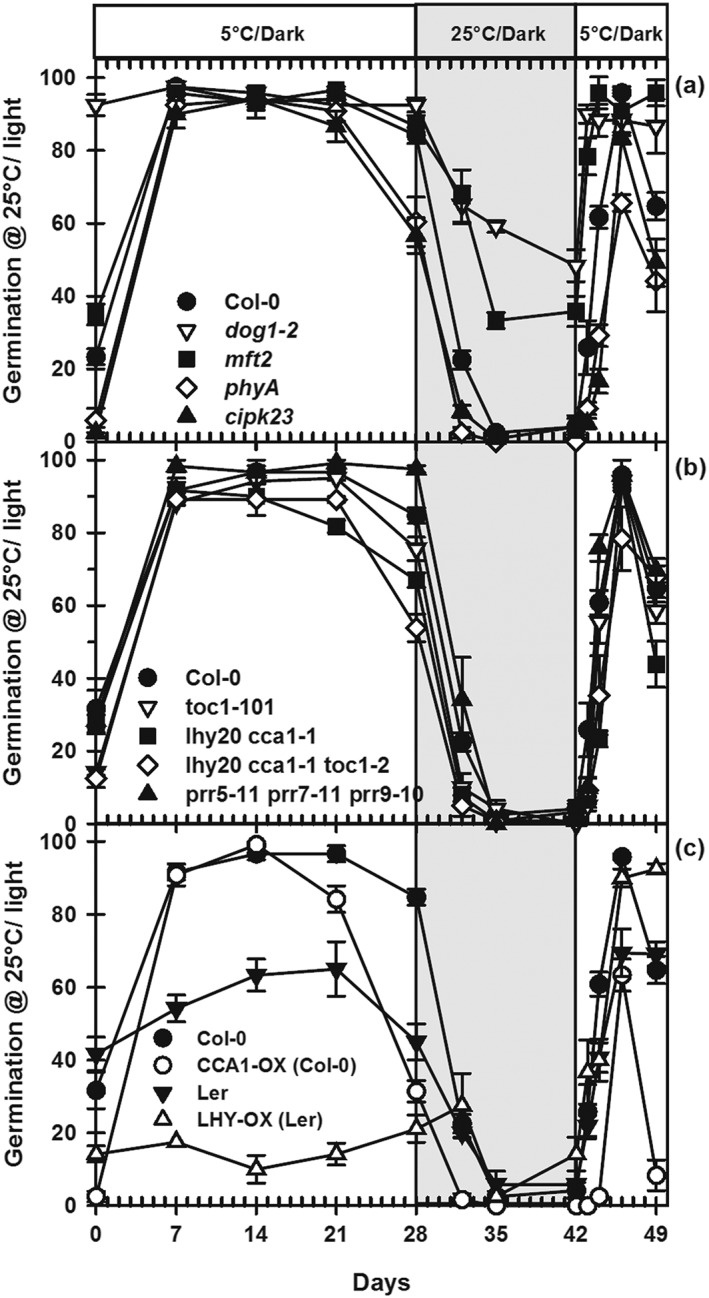
Simulated dormancy cycling in Col‐0, Ler and mutants in dormancy‐related and clock genes. Following 5 °C/dark at −1.0 MPa for 28 d, seeds were transferred to water and incubated in the dark at 25 °C for 14 d before transferring to 5 °C/dark. At increasing intervals, dormancy status was determined by measuring germination on water at 25 °C/light for 14 d. (a) Dormancy‐related mutants, (b) circadian clock mutants and (c) *CCA1* and *LHY* overexpressing lines. Data are mean ± SE (*n* = 3). Absence of error bars indicates that SE is smaller than the symbol.

Col‐0 and Ler seeds were produced by maturing them at the relatively low temperature of 16 °C. This lower temperature increased the level of primary dormancy and prevented dark germination at low temperature. Subsequent imbibition of these seeds at low temperature relieved primary dormancy, and high temperature then induced secondary dormancy (Fig. 3). Dormancy was initially similar in Col‐0 and Ler, but the exposure to low temperature revealed that Ler was the more dormant ecotype (Fig. 3a & c).

To simulate a dormancy cycle in the laboratory, Col‐0 and Ler seeds were first subjected to low temperature (5 °C/dark) at −1.0 MPa for up to 28 d. On day 28, seeds were transferred to water at 25 °C/dark (Fig. 3; results at 25 and 30 °C are shown in Fig. S2, also 20, 25 and 30 °C in Figs S3 & S4), which resulted in the rapid induction of secondary dormancy in all seeds. This was followed by a second low‐temperature phase to relieve secondary dormancy. At all stages, dormancy level was determined by germination following transfer to 25 °C/light. At this temperature, these ecotypes exhibit high‐temperature thermodormancy. Seeds from lines with mutated dormancy‐regulating genes (*dog1‐2*, *mft2*, *cipk23* and *phyA*) and mutated clock genes and overexpressing lines were also subjected to this simulation.

To test if incubation of Col‐0 seeds beyond 28 d at low temperature (5 °C/dark) at −1.0 MPa would induce secondary dormancy, seeds were incubated for up to 42 d. Although primary dormancy was relieved and germination was 94% after 21 d, it only declined to 84% after 28 d and 83% at 42 days indicating a slow induction of secondary dormancy that may increase if the treatment was extended further (see Discussion).

### Dormancy cycling in mutants of dormancy‐related genes

Primary dormancy in the mutants *mft2*, *cipk23* and *phyA* was similar to Col‐0, but *dog1‐2* was non‐dormant (day 0; Fig. 3a). The response of these mutants to the dormancy cycle simulation differed greatly from Col‐0. The initial low‐temperature phase relieved primary dormancy and then induced secondary dormancy in *cipk23* and *phyA* after 21 d. On transfer to higher temperature (25 °C), secondary dormancy induction was complete after only 4 d in *cipk23* and *phyA*, but 7 d in Col‐0 (Fig. 3a). In contrast, *dog1‐2* and *mft2* secondary dormancy induction was slower. Maximum induction was after 14 d in *dog1‐2* (germination 48%) and 7 d in *mft2* (germination 33%). On transfer to the second low‐temperature phase, secondary dormancy was broken after 2 d in *dog1‐2* and *mft2*, and after 4 d in Col‐0, *cipk23* (83%) and *phyA* (65%). Secondary dormancy was then reinduced in Col‐0, *cipk23* and *phyA*, but not in *dog1‐2* and *mft2*. The rate of change was greater when 30 °C was used to induce secondary dormancy, but the relative performance of the lines was very similar (Fig. S2). As *CIPK23* is involved in the regulation of nitrate transport and signalling, the nitrate sensitivity of Col‐0 and *cipk23* was tested when secondary dormancy was induced at 25 °C for 14 d. Germination was 85 and 77%, respectively, in the presence of 10 mm nitrate at 25 °C/light.

To determine the role of the initial cold treatment, seeds were exposed directly to high temperature (25 or 30 °C) in the dark. Secondary dormancy was not induced in *dog1‐2* but was in the wild type and other dormancy‐related mutants (Fig. S5).

### Selection of lines to test whether clock genes influence seed dormancy cycling

We subjected seeds from lines with the following clock mutations: *toc1‐101, lhy20 cca1‐1, lhy20 cca1‐1toc1‐2* and *prr5–11 prr7–11 prr9–10* and the overexpressing lines *LHY‐OX* and *CCA1‐OX* to the dormancy cycling simulation. This combination of mutants allowed us to investigate whether altering the balance between the morning and evening loops of the clock would alter the dormancy cycling response under the relatively long‐term, but changing, constant temperatures of the simulation in the dark (i.e. in the absence of an imposed external daily rhythm).

### Dormancy cycling in clock mutant lines

Primary dormancy of all lines was initially relieved during the low‐temperature phase, but secondary dormancy induction started between days 21 and 28 except in *prr5‐11 prr7‐11 prr9‐10*, and induction increased in the order *toc1‐101, lhy20 cca1‐1 and lhy20 cca1‐1 toc1‐2* (Fig. 3b). On transfer to higher temperature (25 °C) at 28 d, secondary dormancy was completely induced after a further 7 d and was slowest in *prr5‐11 prr7‐11 prr9‐10*. On transfer, back to low‐temperature secondary dormancy was rapidly relieved and then reinduced in all lines (Fig. 3b). The impact of the high‐temperature phase on rate of dormancy induction and its subsequent relief differed with temperature (20, 25 and 30 °C; Fig. S3).

The overexpressing lines behaved differently from their respective wild‐type comparisons. In the *CCA1‐OX* (Col‐0 background), overexpressing line secondary dormancy was more rapidly induced during the first low‐temperature phase than in Col‐0 and was complete after only 4 d on transfer to 25 °C compared with 7 in Col‐0 (Fig. 3c), whereas in the *LHY‐OX* (Ler background), overexpressing line was more dormant than Ler, and the first low‐temperature phase did not relieve dormancy. Dormancy increased on transfer to 25 °C (Fig. 3c). In the second low‐temperature phase, secondary dormancy was relieved but rapidly reinduced only in *CCA1‐OX*. The response to the second low‐temperature phase was dependent on the previous temperature. For example, in contrast to that shown on transfer from 25 °C (Figs 3b & S4b), on transfer from 20 °C secondary dormancy was not relieved by low temperature in *CCA1‐OX* (Fig. S4a). Furthermore, on transfer from 30 °C to low‐temperature secondary dormancy was relieved but only reinduced with prolonged incubation (Fig. S4c).

### Response of clock genes to temporal signals in the field

**Figure 4:**
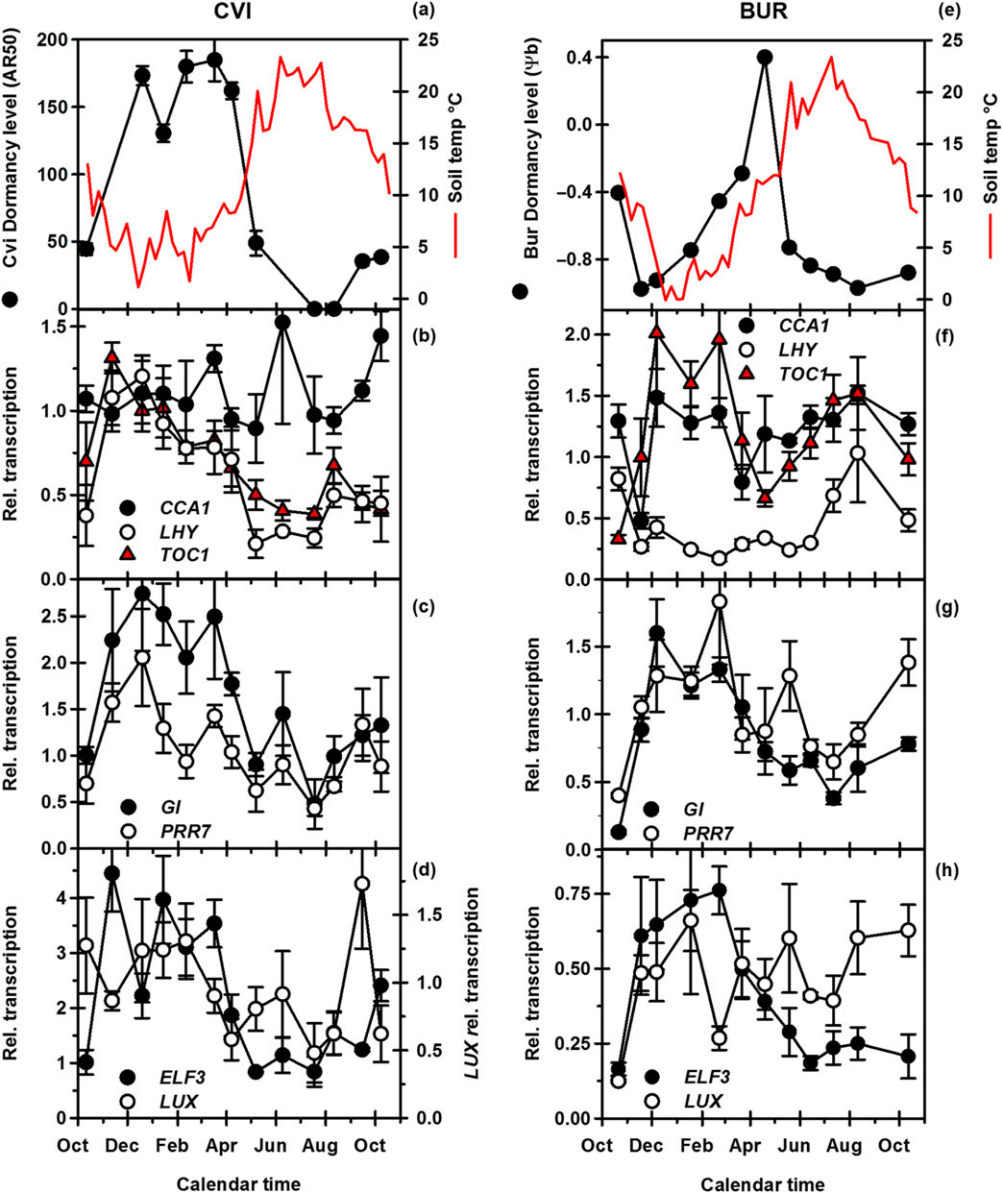
Seasonal coordination of clock gene transcription in winter (Cvi) and summer annual (Bur) ecotypes. Depth of dormancy in (a) Cvi [time to 50% after‐ripening (AR50)] and (e) Bur [base water potential (Ψb)] with soil temperature at seed depth (data from Footitt *et al*. 2011, 2013). Transcription profiles of the morning genes *CCA1* and *LHY* and the evening gene *TOC1* in (b) Cvi and (f) Bur. Transcription profiles of *GI* and *PRR7* in (c) Cvi and (g) Bur. Transcription profiles of evening complex genes *ELF3* and *LUX* in (d) Cvi and (h) Bur. Cvi, Cape Verde Island; Bur, Burren.

To further understand the response of clock mutants, we analysed the transcription profiles of selected clock genes in seeds of the deeply dormant winter annual ecotype Cvi and the shallow dormant summer annual ecotype Bur during dormancy cycling in the field (Fig. 4). Bur is a summer annual ecotype whose dormancy cycling behaviour is highly characterized (Footitt *et al.* 2013, 2015). As such, it is used here as a model for the summer annual behaviour of the ecotype Col‐0, the genetic background of the clock mutants used in the laboratory simulation. In both Cvi and Bur ecotypes, there were clear annual transcript profiles; however, the profiles of the morning genes *CCA1* and *LHY* differed between ecotypes (Fig. 4b & f). In Cvi, the transcription profiles of *LHY* and *TOC1* were similar, but opposite to the soil temperature profile, whereas in Bur, *CCA1* and *TOC1* transcript profiles are similar but have little relationship with the temperature profile (Table S1). In Cvi and Bur, *GI*, *PRR7* and *ELF3* transcription profiles are the same and inversely tracked soil temperature and in the case of Cvi also tracked dormancy (Fig. 4c, d, g & h). Of the evening complex genes examined, *LUX* transcription had no obvious pattern in contrast to *ELF3* (Fig. 4d & h).

Significant correlations occurred between the transcription profiles of the clock and dormancy‐related genes and the annual soil temperature cycle in both ecotypes (Table S1). In particular, there were strong correlations between the evening genes *ELF3* and *GI* and chromatin remodelling genes involved in gene activation/repression such as *HUB1* and *OTLD1* and silencing (*KYP*/*ROS1*) (Footitt *et al.* 2015) (Table S1).

### Dormancy induction and thermal time

**Figure 5:**
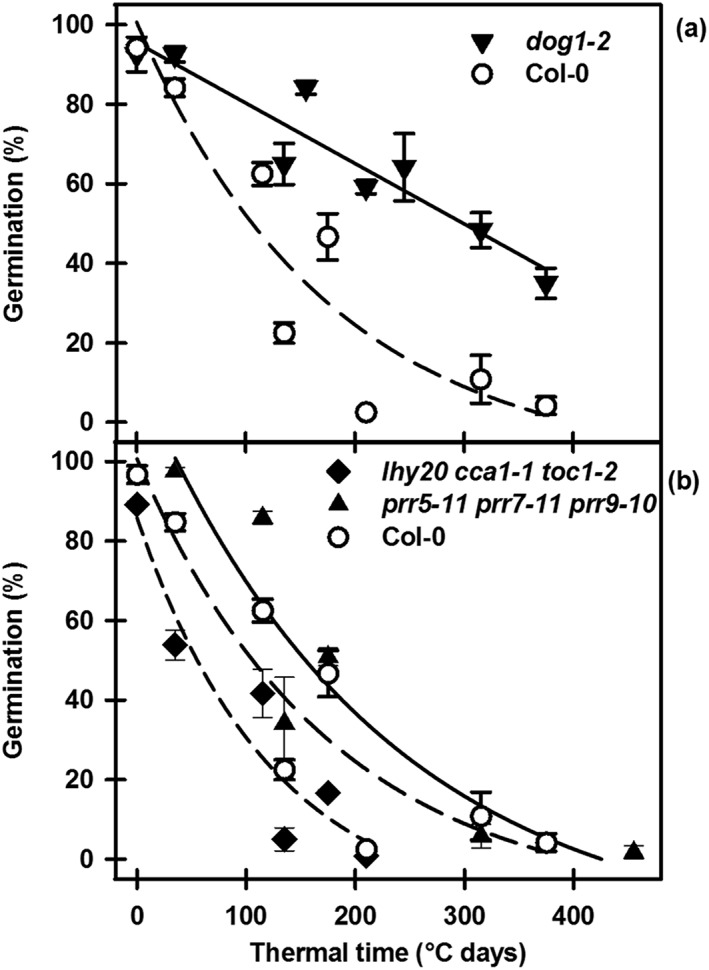
Thermal time analysis of dormancy induction at high temperature following low‐temperature conditioning. (a) Wild type (Col‐0) and the dormancy mutant, *dog1‐2*, and (b) wild type (Col‐0) and the circadian clock mutants, *lhy20 cca1*‐1 *toc1‐2* and *prr5‐11 prr7‐11 prr9‐10*. Data from Figs 3, S1 and S2 are replotted against thermal time (sum of temperature above 0 °C) for secondary dormancy induction at 20, 25 and 30 °C. The response to thermal time fits the following relationships: exponential decay (three parameters) regressions describe Col‐0 (*R* ^2^ = 0.972), *lhy20 cca1‐1 toc1‐2 (R* ^2^ = 0.897) and *prr5‐11 prr7‐11 prr9‐10* (*R* ^2^ = 0.860), while a linear regression describes *dog1‐2* (*R* = 0.928). The same data for Col‐0 appear in (a) and (b).

In the field, dormancy induction and relief during cycling were shown to progress in thermal time (Footitt *et al*. 2011). We therefore used this approach to analyse data in the laboratory simulation during dormancy induction at 20, 25 and 30 °C (Figs 5 & S6). Induction of secondary dormancy in Col‐0, *mft2* and the clock mutants followed an exponential decay response with thermal time. In the clock mutants, the thermal time required to induce secondary dormancy in 50% of the population decreased in the order *prr5*‐*11 prr7‐11 prr9‐10* (153 °C d) > Col‐0 (105 °C d) > *toc1‐101* (82 °C d) > *lhy20 cca1‐1* (60 °C d) > *lhy20 cca1‐1 toc1‐2* (54 °C d). In *dog1‐2*, induction of secondary dormancy had a linear response (see Fig. S6 for regression equations).

### Abscisic acid sensitivity of Col‐0, dormancy and clock mutants

**Figure 6:**
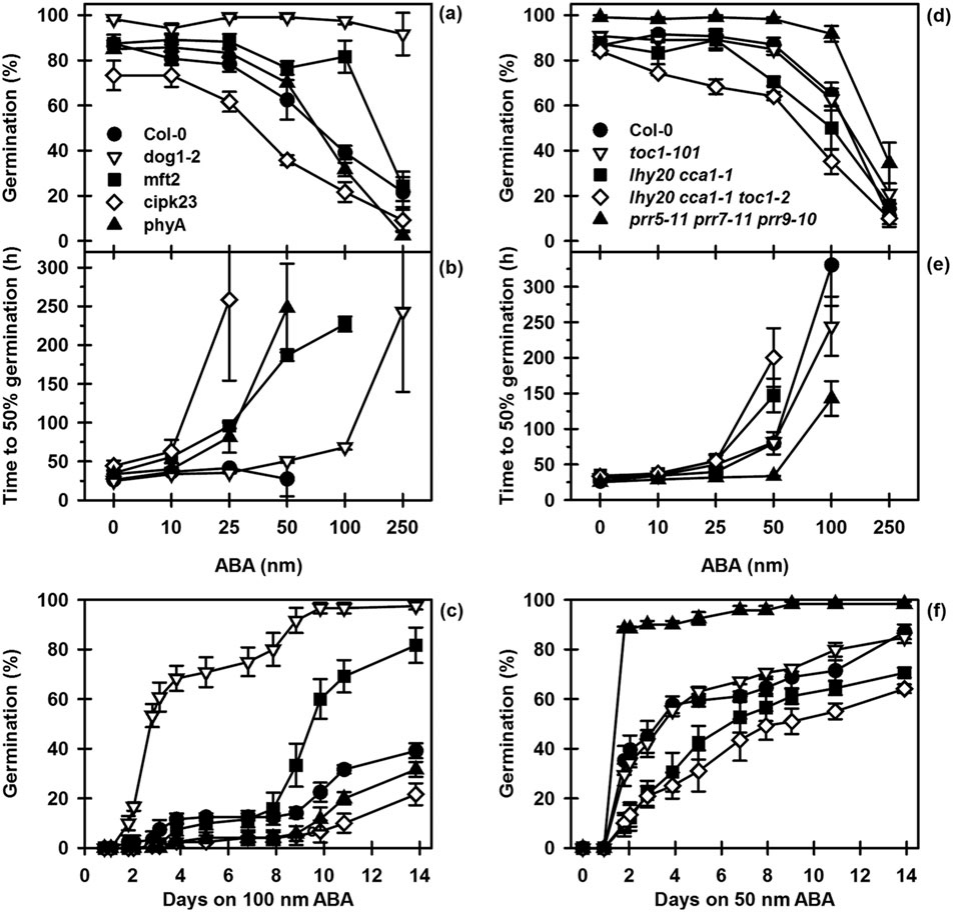
ABA sensitivity of dormancy and clock mutants. Following 3 d at 5 °C/dark on water seeds were transferred to ABA (10–250 nm) in buffer at pH 5.0, and cumulative germination was recorded during incubation at 25 °C/light over 14 d. Final germination at each concentration after 14 d (a) and (d). The time to 50% germination (b) and (e) in hours (h) of data in (a) and (d), respectively. Cumulative germination of dormancy mutants in the presence of 100 nm ABA (c). Cumulative germination of clock mutants in the presence of 50 nm ABA (f). Data are mean ± SE (*n* = 3). Absence of error bars indicates that SE is smaller than the symbol. ABA, abscisic acid.

Because of the role of ABA in the induction of dormancy, we investigated ABA sensitivity of both groups of mutants. Dormancy mutants showed large differences in ABA sensitivity. With the exception of *dog1‐2*, final germination was similar in all lines (Fig. 6a). However, the speed of germination represented by the time to 50% germination (T50) (a measure of germination velocity) revealed that ABA sensitivity increased in the order *dog1‐2* > *mft2* > Col‐0 > *phyA* > *cipk23* (Fig. 6b) similar to that seen for the induction of secondary dormancy in thermal time (Figs 5a & S6a). The response of dormancy mutants to 100 nm ABA (Fig. 6c) illustrates further their different ABA sensitivities. ABA sensitivity in *dog1‐2* was greatly reduced in agreement with that reported for *dog1‐1* (Ler background) (Bentsink *et al.* 2006). The onset of low ABA sensitivity was delayed in *mft2* potentially indicating delayed ABA catabolism as an uplift in germination that occurred at the same time in all lines (Fig. 6c).

Clock mutants also exhibit different ABA sensitivities (Fig. 6d). The time to 50% germination (T50) revealed that ABA sensitivity increased in the order *prr5*‐*11 prr7‐11 prr9‐10* > *toc1‐101* > Col‐0 > *lhy20 cca1‐1* > *lhy20 cca1‐1 toc1‐2* (Fig. 6e) again similar to secondary dormancy induction in thermal time (Fig. 5b). The response to 50 nm ABA indicates that these differences are constant during germination (Fig. 6f). The overexpressing lines have similar ABA sensitivity to their parental wild types (Fig. S7).

### Germination in the dark

Germination is reported following transfer to the light including the limited dark germination. Dark germination was also recorded to determine if temperature manipulation replaced dormancy relief by light. There was no dark germination in the first low‐temperature phase. In the high‐temperature phase, dark germination in the dormancy mutants peaked at 25 in *dog1‐2* and 32% in *mft2* at 25 °C, and 9 and 2% at 30 °C (Fig. S8). In the clock mutants and overexpressers, dark germination was 5% or less at 20 and 25 °C (Fig. S9) with none at 30 °C. In Col‐0, maximum dark germination at high temperature was 11% (Figs S8 & S9). In the second low‐temperature phase, dark germination shows little response. In *cipk23* and *phyA*, maximum dark germination was 2%.

## Discussion

Gene expression patterns during annual dormancy cycling in the field can differ from those anticipated from more static laboratory dormancy studies (Footitt *et al*. 2011, 2013; Finch‐Savage and Footitt 2017). We therefore established a robust laboratory simulation of dormancy cycling in different *Arabidopsis* ecotypes by manipulating temperature and water potential. The central role of temperature in dormancy cycling is well known (Probert 2000), and the role of low water potential on the induction of secondary dormancy in the dark was originally shown by Khan & Karssen (1980). Furthermore, primary dormancy status upon shedding is known to influence subsequent cycling; for example, it can impact on the induction of secondary dormancy by low water potential in Col‐0 (Auge *et al*. 2015). In the experiments presented, ecotypic differences in the relief and induction of dormancy by the temperatures used in the simulation were consistent with those previously shown for Bur, Col‐0, Ler and Cvi (Cone and Spruit 1983; Huang *et al.* 2015, Springthorpe and Penfield 2015; Penfield & Springthorpe 2012). These differences presumably arose during adaptation to their specific climates from a common underlying species response. This adaptation occurs in the initial depth of primary dormancy and the subsequent balance of induction and relief so that cycling behaviour may differ within and between ecotypes if the environment changes.

Initial depth of dormancy is determined by both genetics and environmental exposure pre‐shedding and post‐shedding (Finch‐Savage and Footitt 2017). The effect of the latter is illustrated here in data from the seeds of the winter annual ecotype Cvi from the same harvest, but with different depths of dormancy resulting from post‐harvest conditions. Seeds with greater depth of dormancy (Fig. 1) did not become light sensitive upon exposure to low temperature but became more dormant, whereas a proportion of seeds in a less dormant seed lot (Fig. 2) became light sensitive before the whole seed population subsequently became more dormant on continued exposure. Cycling behaviour therefore differed depending on the environmentally determined initial depth of dormancy. In both seed lots, dormancy in Cvi was then relieved by exposure to higher temperatures (Footitt *et al*. 2011; Huang *et al*. 2015). This is apparently in direct contrast to the results with the summer annual ecotypes Bur, Col‐0 and Ler in which increasing temperature accelerated the induction of secondary dormancy (Fig. 3; Cone and Spruit 1983; Huang *et al.* 2015, Springthorpe and Penfield 2015). However, Col‐0 can behave as both a winter and summer annual in the field (Springthorpe and Penfield 2015) suggesting secondary dormancy may also be relieved by high temperature as seen in Cvi depending on the environment before and after shedding. Thus in Col‐0, further induction of secondary dormancy by low temperature may be required before a change to high temperature results in relief. However, this intriguing aspect of dormancy cycling in Col‐0 is yet to be demonstrated.

The aforementioned results raise the question of how dormancy cycling is driven by temperature and time (thermal time) to alter the balance between induction and relief of dormancy as part of a dormancy continuum. In this continuum, as primary dormancy in the dispersed seed is relieved, in response to the prevailing environmental conditions (predominantly temperature), the same conditions will start to induce secondary dormancy if the environmental signals required to remove the final layer of dormancy are not received. This behaviour is consistent with the hypothesis that temperature impacts the rate of dormancy induction and relief independently but importantly that these processes may occur simultaneously (Totterdell and Roberts 1979; Batlla *et al*. 2009). These opposing processes are largely governed by the environmental sensitivity of the ABA/GA hormone balance (Finch‐Savage and Leubner‐Metzger 2006; Finch‐Savage and Footitt 2017). Initial primary dormancy level determines the temperature sensitivity of both induction and relief via changes in this balance. The hypothesis implies that the terms primary and secondary dormancy are only descriptive of sequences in the cycle with no physiological relevance as dormancy is a continuum, and only the level changes.

The protocol presented could be used to test this hypothesis and further develop our understanding of dormancy cycling by evaluating responses of different ecotypes to temperature and water potential. The ecotypes used in the present work included Col‐0 as the common genetic background for 1000s of mutant lines (http://arabidopsis.info/) to facilitate genetic dissection of dormancy cycling. We discuss later how the protocol also has a great potential as an investigative tool in advancing our understanding of the role of genes in dormancy regulation.

### Regulation of dormancy cycling

How ABA and GA signalling pathways are coordinated during dormancy cycling by temperature and water potential is not fully understood (Finch‐Savage and Footitt 2017). It was argued previously (Footitt *et al.* 2013) that changing temporal signals linked to the transcription of *DOG1*, *MFT*, *PHYA* and *CIPK23* drives regulation of dormancy cycling. *DOG1* and *MFT* expression contributes to thermal time sensing linked to changes in *CIPK23* and *PHYA* expression that results in altered sensitivity to spatial signals (nitrate and light, respectively) indicating suitability for germination. The data presented for mutants of these genes subjected to the laboratory simulation of dormancy cycling (Fig. 3) support the correlative observations made in the field. Thermal time analysis showed that dormancy induction in the absence of *DOG1* (*dog1‐2*) was linear with thermal time and exponential in its presence (Fig. 5). This adds to the contention that *DOG1* is part of a thermal mechanism sensing in an annual seasonal pattern (circannual rhythm) and may amplify thermal signals by increasing ABA sensitivity. Mutants in *CIPK23* and *PHYA* show an increased induction of secondary dormancy. Both *PHYA* and *CIPK23* also influence hormone signalling consistent with the importance of the dynamic ABA/GA balance determining dormancy levels in response to environmental signals (Finch‐Savage and Footitt 2017).

### DELAY OF GERMINATION 1 and MOTHER OF FLOWERING TIME

During seed development, *DOG1* is absolutely required for the induction of dormancy (Dekkers *et al.* 2016). However, in *dog1‐1* (Ler background), low dark germination was seen in fresh seeds that could be removed by low temperature indicating that a low level of primary dormancy was present at maturity (Bentsink *et al.* 2006). In the dormancy simulation, high temperature alone did not induce secondary dormancy in *dog1‐2* as it had high levels of dark germination followed by full germination on transfer to light (Fig. S5). However, cold pre‐conditioning at −1 MPa induced a low level of secondary dormancy at the end of the initial cold phase. Light was increasingly unable to remove the final layer of dormancy in Col‐0 but not *dog1‐2* (Fig. 3a, days 21–28). This small loss of sensitivity to light indicates that in the Col‐0 genetic background secondary dormancy induction was starting to dominate its relief. On transfer to the higher temperature, this level of secondary dormancy was sufficient to prevent dark germination in *dog1‐2* as well as in Col‐0, while dormancy induction increased to the point where seeds were no longer light sensitive (Fig. 3). On the basis that any environmental signal that widens the conditions required for germination is in effect altering dormancy (Finch‐Savage and Footitt 2012), we conclude that the induction of a light requirement and the decreasing sensitivity to light with increasing thermal time is evidence for the induction of secondary dormancy in *dog1‐2*. This indicates that these conditions allow other factors to impose secondary dormancy in the absence of *DOG1.* One potential candidate is *MFT*.

Induction of secondary dormancy in thermal time was slower in *dog1‐2* than in *mft2* indicating the primacy of *DOG1* over *MFT* during dormancy induction. Further research is required to confirm a role for *MFT* in thermal sensing. The greatly reduced induction of dormancy in *dog1‐2* is consistent with DOG1 amplifying thermal signals via increased sensitivity to ABA. The dramatically lower ABA sensitivity of *dog1‐2* reported here supports this (Fig. 6a–c).

The loss of ABA sensitivity in *mft2* shows that MFT contributes positively to ABA signalling (Fig. 6). This is via the oxylipin, 12‐oxo‐phytodienoic acid (OPDA), which acts through MFT to induce ABA biosynthesis and sensitivity (Dave *et al.* 2016). Then MFT and ABA via a feedback loop enhance OPDA levels further contributing to DOG1 germination repression (Dave *et al.* 2016) explaining the ABA hypersensitive germination of *MFT* overexpressing lines (Hu *et al.* 2016). The delayed response to ABA compared with *dog1‐2* may reflect declining ABA levels when the OPDA pathway is blocked. In contrast, fully after‐ripened *mft2* seeds are ABA hypersensitive (Xi *et al.* 2010). This may reflect a changing temporal sensing role for *MFT* dependent on ecotype and the seasonal onset of the dormancy cycle as reflected in altered timing of *MFT* transcription in the field (Footitt *et al.* 2013, 2014). This role for MFT in shallow dormancy when DOG1 levels are low is discussed elsewhere (Finch‐Savage and Footitt 2017).

During the final low‐temperature phase, secondary dormancy is broken faster in *dog1‐2* and *mft2*, than in the wild type. Low‐temperature treatment then reinduced secondary dormancy in the wild type but not in these mutants showing that dormancy cycling at low temperature is compromised.

### PHYTOCHROME A and CBL‐INTERACTING PROTEIN KINASE 23

Unlike *mft2* and *dog1‐2*, secondary dormancy was induced in *phyA* and *cipk23* by low temperature, which then accelerated on transfer to higher temperatures (Figs 3a & S2). This induction of secondary dormancy at high temperature, its relief and reinduction in the second low‐temperature phase is consistent with increased ABA sensitivity compared with Col‐0. This is supported by the ABA hypersensitivity of *cipk23* (Fig. 5a–c). The limited ABA response of *phyA* reflects the increased contribution of other negative regulators of germination potential in this mutant (Ibarra *et al.* 2013).

### PHYTOCHROME A

Phytochrome A is responsible for the very low fluence response whereby the final layer of dormancy is removed by brief exposure to light during soil disturbance (Batlla and Benech‐Arnold 2014). The increased sensitivity of *phyA* seeds to temperature and water stress is consistent with enhanced ABA sensitivity (Figs 3a and 6a–c). Transcriptome comparisons between wild‐type and *phyA* seeds support this with 11% of the expressed transcriptome significantly regulated by PHY*A* (Ibarra *et al.* 2013). Of those significantly up‐regulated by PHY*A*, 7% are transcription factors linked with auxin and GA responses, and ABA catabolism, while down‐regulated genes contain representatives of the ABA signalling pathways and *DELLA* genes that relieve repression of GA signalling (Ibarra *et al.* 2013). So in *phyA* seeds, the balance of the ABA/GA signalling pathways favours ABA amplifying the response to dormancy‐inducing temporal signals.

### CBL‐INTERACTING PROTEIN KINASE 23

This protein forms a calcium‐sensing complex with CBL1 or CBL9 (CALCINEURIN B‐LIKE PROTEIN), which is involved in iron, nitrate and potassium transport and sensing (Léran *et al*. 2015; Manik *et al.* 2015; Tian *et al.* 2016). Its role in regulating nitrate transport and signalling by the NITRATE TRANSPORTER 1.1 (NRT1.1) transceptor (duel nutrient transport/signalling function) and the crucial role this plays in the regulation of ABA levels in seeds is well characterized (reviewed in Finch‐Savage and Footitt 2017).

The ABA hypersensitivity of *cipk23* seeds (Fig. 6a–c) indicates that ABA signalling is enhanced in the absence of CIPK23. In the field, low dormancy is coincident with increased nitrate sensing, which is preceded by enhanced *NRT1.1* expression and reduced *CIPK23* expression. The subsequent onset of secondary dormancy induction appears to reduce nitrate signalling below threshold levels both by reducing the amount of NRT1.1 and its phosphorylation via CIPK23‐CBL1/9 (reviewed in Finch‐Savage and Footitt 2017; Footitt *et al.* 2011, 2013, 2014). Here, Col‐0 and *cipk23* lose light but not nitrate sensitivity during induction of secondary dormancy suggesting that loss of nitrate sensitivity is related to NRT1.1 protein levels and the action of factors such as DOG1 that regulate deep dormancy (reviewed in Finch‐Savage and Footitt 2017). CIPK23‐CBL complexes also have other functions, for example, as nutrient sensors to monitor mineral homeostasis in general (Tian *et al.* 2016). Further work is therefore needed to fully understand the role of *CIPK23* in dormancy regulation.

### Dormancy regulation and clock genes

The annual seasonal rhythm of soil temperature (Fig. 6a and b) was correlated with transcriptional responses of the dormancy‐related genes discussed previously (Table S1). During seasonal bud dormancy regulation in trees, perception of seasonal temperature signals involves components of the circadian clock (Cooke *et al.* 2012). We tested if this could also be occurring in seeds by analysis of clock mutants in the laboratory simulation and by measuring clock gene expression over an annual cycle in the field. The results obtained were consistent with the balance between the evening and morning phases of the clock contributing to the interpretation of temperature signals (thermal time) that determine cycles of dormancy induction and relief.

### Clock mutants in the laboratory simulation

In this series of laboratory simulations, successive relatively long‐term incubations at constant temperatures in the dark show that the clock has an impact on dormancy status without an imposed external daily rhythm (Figs 3, S2, S3 & S4). In the parental wild type (Col‐0), secondary dormancy was induced on transfer to high temperature and increased further as temperature was raised (20 > 25 > 30 °C). Lines with mutations in the morning genes *LHY* and *CCA1* (*lhy20 cca1‐1* and *lhy20 cca1‐1 toc1‐2*) had the highest ABA sensitivity and the most rapid induction of secondary dormancy, whereas the triple mutant *prr5*‐*11 prr7‐11 prr9‐10* had the lowest ABA sensitivity and slowest induction (Fig. 6d–f). This disruption of the morning loop by mutations in *LHY* and *CCA1* would reduce repression of the evening loop genes *TOC1* and *GI* and the evening complex genes *LUX*, *ELF3* and *ELF4* (Pokhilko *et al*. 2013). The *prr5*‐*11 prr7‐11 prr9‐10* mutant would reduce repression of *LHY* and *CCA1*. Therefore, this result indicates a critical balance between the morning and evening signalling components if the clock influences the induction of dormancy. It further implies that in the absence of a fully functioning morning loop, repression of *TOC1*, *GI* and the evening complex genes is incomplete. This is consistent with observations of delayed bud burst (loss of dormancy) in *Populus LHY* mutants (Ibáñez *et al*. 2010). These data are also consistent with clock gene transcription recorded during the annual soil temperature cycle in the field (Fig. 4) and are discussed later. These field data indicate that the annual seasonal cycle is analogous to an extended diurnal cycle with low winter temperatures representing the evening phase and summer temperatures representing the morning phase (circannual dormancy rhythm). Thermal time analysis (Fig. 5) shows that dormancy cycling responds to the strength of the inductive thermal time signal generated by the clock.

### Annual clock gene expression in the field

We followed gene expression in the contrasting ecotypes Bur (summer annual) and Cvi (winter annual).The transcript profiles of evening genes increased with falling temperature and therefore in general were negatively correlated to the annual soil temperature cycle in both ecotypes (Table S1). Surprisingly, the morning genes *LHY* (in Cvi) and *CCA1* (in Bur) have the same transcript profiles as *TOC1*, while only *LHY* transcription in Bur correlates positively with temperature. This contrasts the general situation in the clock where the transcript profile of *TOC1* is in the opposite phase to both *LHY* and *CCA1* (Salome and McClung 2005; Gould *et al.* 2006). However, it is consistent with high transcription of *TOC1* and *LHY* in chestnut internodes during winter when the clock becomes arrhythmic (Ibañez *et al*. 2008). Notably *LHY* (in Cvi) and *CCA1* (in Bur) transcription does not return to the opposite phase of *TOC1* in the warm summer months. It is also notable that in Bur, *TOC1* transcription also increases with summer temperature and at that point is similar to both *LHY* and *CCA1*. This suggests that adaptation of dormancy cycling to the environment may involve allelic variation in clock genes as seen in *Drosophila* (Yamada and Yamamoto 2011).

Components of the clock will alter the central integrating ABA/GA balance controlling dormancy cycling. *TOC1* and the clock are involved in the gating of ABA responses (Seung *et al.* 2012). *TOC1* is induced by ABA and interacts with genes involved in ABA signalling responses (Seung *et al.* 2012). In addition, it interacts with the positive regulator of dormancy *ABA* *INSENSITIVE 3* (*ABI3*) (Kurup *et al.* 2000). *ABI3* mutants also exhibit altered circadian rhythms (Pearce 2003). The consequence of increased *TOC1* transcription therefore appears to be an up‐regulation in ABA signalling. GA biosynthesis is repressed by the evening loop with increased expression of the GA biosynthesis gene *GA20OX2* found in *toc1*, and increased levels of bioactive GA and *GA20OX2* found in *elf3* (Atamian and Harmer 2016). This again indicates evening loop involvement in dormancy cycling.

Dormancy and ABA levels initially increase together, but a point is reached where dormancy increases are ascribed to increasing ABA sensitivity via DOG1 (Footitt *et al.* 2011). Interestingly, circadian rhythm microarray data from Col‐0 seedlings (Edwards and Millar 2007) show rhythmic *DOG1* transcription (http://bar.utoronto.ca/efp/cgi‐bin/efpWeb.cgi?dataSource=Light_Series) (Fig. S10). In contrast, morning gene transcription is more positively correlated with genes up‐regulated in the spatial sensing phase of the dormancy cycle (Table S1).

### Circannual dormancy rhythm

Annual cycling of the depth of dormancy is well documented (Baskin and Baskin 1998), and understanding of how this is regulated by a range of dormancy mechanisms in response to environmental signals is developing (Finch‐Savage and Footitt 2017). These mechanisms operate via a central integrating ABA/GA balance to time germination completion in the optimum season and habitat. Here, we confirm the key involvement of *DOG1*, *MFT*, *CIPK23* and *PHYA* in regulating the depth of dormancy. Furthermore, we show based on mutant analyses and transcript profiles that the balance between the evening and morning phases of the clock also reflects this circannual dormancy rhythm. Based on the thermal time and ABA sensitivity data, dormancy cycling appears to respond to the strength of the inductive thermal time signal generated by the clock. Further directed research is required to test these hypotheses and provide details of the clock's involvement. Nevertheless, circannual rhythms for germination timing are seen in seeds of the desert annual Mesembryanthemum nodiflorum and in cysts of the marine dinoflagellate *Alexandrium* in constant conditions over several years (Gutterman and Gendler 2005; Matrai *et al*. 2005) and may be part of a bet‐hedging strategy. How a circannual clock contributes to and maintains annual rhythms over several years is unclear.

## Author Contributions

S. F. and W. E. F.‐S. designed the research. H. Ö.‐F. performed Cvi experiments. S. F. and A. J. H. performed all other experiments. All authors analysed the data. S. F and W. E. F‐S. wrote the manuscript.

## Supporting information

Data S1. Materials and methods

Table S1. Correlation table

Table S2. Primers

Figure S1. Box layout for incubation of seeds at reduced water potential.

Figure S2. Simulated dormancy cycling in dormancy related mutants at 25°C and 30°C.

Figure S3. Simulated dormancy cycling in clock mutants at 20°C, 25°C and 30°C.

Figure S4. Simulated dormancy cycling in *CCA1* and *LHY* overexpressing lines at 20°C, 25°C and 30°C.

Figure S5. Response of dormancy related mutants when placed directly in high temperature without cold conditioning at low water potential.

Figure S6. Thermal time analysis of dormancy induction at high temperature following low temperature conditioning of the dormancy mutants' *dog1–2* and *mft2* and clock mutants.

Figure S7. ABA sensitivity of Col‐0 and Ler wild types and *CCA1* and *LHY* overexpressing lines.

Figure S8. Dark germination of Col‐0, and the dormancy mutants *dog1–2* and *mft2*.

Figure S9. Dark germination of Col‐0, clock mutants and *CCA1* and *LHY* overexpressing lines.

Figure S10. *DOG1* transcript level in Col‐0 seedlings entrained to a light/dark cycle.
