# Supplementary material for: A laboratory simulation of Arabidopsis seed dormancy cycling provides new insight into its regulation by clock genes and the dormancy‐related genes DOG1, MFT, CIPK23 and PHYA
Source: Plant Cell Environ. 2017 May 16;40(8):1474–86. doi: 10.1111/pce.12940 (PMC5518234; doi:10.1111/pce.12940)
Supplement: Supplementary file 1 — Data S1. Materials and methods [file PCE-40-1474-s011.docx]

**SI Text**

**Materials and Methods**

**Seed production in wild type Ler, Col-0 and mutants:** Seeds were surface sterilised in 2.5% dilution of domestic bleach for 5 minutes, washed three times in water, plated out using Ultipette™ BARKY CP-100 tips (Barkey Instruments International, UK) on to ½ MS salts and 0.7% agarose (pH 5.8), stratified for 3 days at 5°C/dark and then transferred to 20°C in the light. At the first true leave stage, seedlings were transferred to growth medium (Levingtons F1 compost: sand: vermiculite 6:1:1) in P24 cellular trays (24 cells, each 5 x 5 x 5 cm) placed in a second tray lined with capillary matting to ensure plants had a uniform water supply. One tray of each mutant and wildtype plants were incubated at 22°/18°C 16h L/8 h D. When all plants in a tray had bolted (1 cm bolt) all siliques were removed. Each plant was then covered with an Aracon (Betatech bvba, Belgium) and plants transferred to 16°/16°C 16h L/8 h D for subsequent growth and seed production. Seeds were harvested at maturity by hand threshing and cleaned seeds equilibrated at 55% RH/ 20°C for 7 days to produce an equilibrium moisture content of 6-10 % on a dry weight basis. Seeds were then stored at -80°C until required.
