# Supplementary material for: A laboratory simulation of Arabidopsis seed dormancy cycling provides new insight into its regulation by clock genes and the dormancy‐related genes DOG1, MFT, CIPK23 and PHYA
Source: Plant Cell Environ. 2017 May 16;40(8):1474–86. doi: 10.1111/pce.12940 (PMC5518234; doi:10.1111/pce.12940)
Supplement: Supplementary file 3 — Table S2. Primers [file PCE-40-1474-s013.doc]

**Table S2. Primers used for QPCR of field samples.**

| Gene |  | Forward primer | Reverse primer |
| --- | --- | --- | --- |
| At4g34270 | *TIP41-like* | GTGAAAACTGTTGGAGAGAAGCAA | TCAACTGGATACCCTTTCGCA |
| At4g12590 |  | GAGATGAAAATGCCATTGATGAC | GCACCCAGACTCTTTGATG |
| At5g61380 | *TOC1* | AGTCACCAGGAAAATGAGTGG | AAGACCACCATCACGAGCAT |
| At2g46830 | *CCA1* | AAAAAGTGTCGCATCCTGAGA | GAACAGTTGTCTTCCTGCAGAGT |
| At1g01060 | *LHY* | AAGAGCTTGGCAACGAATTG | AGCCTCTTTCTCCAACTTTGTG |
| At1g22770 | *GI* | TTTCCGATGGTGTAGTGGTG | TTGAAGGCATCAGTTGAGGA |
| At5g02810 | *PRR7* | GAGTTTCCAAGTAATCAGTTGGTTG | CTCTACCCATTGTGACATCTTCA |
| At2g25930 | *ELF3* | TCGATTTCGTTTTCTCTGATTG | CCCAAAGGACAAACTACGATAGA |
| At3g46640 | *LUX* | ATGGGGGAGGAAGAGATAGG | TTGTATGATCCTCTCCTGAACAGA |
