# Supplementary material for: A laboratory simulation of Arabidopsis seed dormancy cycling provides new insight into its regulation by clock genes and the dormancy‐related genes DOG1, MFT, CIPK23 and PHYA
Source: Plant Cell Environ. 2017 May 16;40(8):1474–86. doi: 10.1111/pce.12940 (PMC5518234; doi:10.1111/pce.12940)
Supplement: Supplementary file 5 — Figure S2. Simulated dormancy cycling in dormancy related mutants at 25°C and 30°C. [file PCE-40-1474-s002.docx]

**Figure S2. Simulated dormancy cycling in dormancy related mutants at 25°C and 30°C.** Following 5°C/dark at -1.0 MPa for 28 days seeds were transferred to water and incubated in the dark at (a) 25°C and (b) 30°C for up to 14 days before transferring to 5°C/dark. At increasing intervals dormancy status was determined by transferring seeds to 25°C/light. Final germination was determined after 14 days. The data in (b) is taken from Fig. 3(a). Data are mean ± SE (n = 3). Absence of error bars indicates SE is smaller than the symbol.
