# Supplementary material for: A laboratory simulation of Arabidopsis seed dormancy cycling provides new insight into its regulation by clock genes and the dormancy‐related genes DOG1, MFT, CIPK23 and PHYA
Source: Plant Cell Environ. 2017 May 16;40(8):1474–86. doi: 10.1111/pce.12940 (PMC5518234; doi:10.1111/pce.12940)
Supplement: Supplementary file 8 — Figure S5. Response of dormancy related mutants when placed directly in high temperature without cold conditioning at low water potential. [file PCE-40-1474-s005.docx]

**Figure S5. Response of dormancy related mutants when placed directly in high temperature without cold conditioning at low water potential.** Data shows dark germination at (a) 25°C and (b) following transfer to 25°C/light for 14 days. Dark germination at (c) 30°C and (d) following transfer to 25°C/light for 14 days. Data are mean ± SE (n = 3). Absence of error bar indicate SE is smaller than the symbol.
