# Supplementary material for: A laboratory simulation of Arabidopsis seed dormancy cycling provides new insight into its regulation by clock genes and the dormancy‐related genes DOG1, MFT, CIPK23 and PHYA
Source: Plant Cell Environ. 2017 May 16;40(8):1474–86. doi: 10.1111/pce.12940 (PMC5518234; doi:10.1111/pce.12940)
Supplement: Supplementary file 9 — Figure S6. Thermal time analysis of dormancy induction at high temperature following low temperature conditioning of the dormancy mutants' dog1–2 and mft2 and clock mutants. [file PCE-40-1474-s006.docx]

**Figure S6.** **Thermal time analysis of dormancy induction at high temperature following low temperature conditioning of the dormancy mutants’ *dog1-2* and *mft2* and clock mutants.** (a) Wild type (Col-0) and the dormancy mutants, *dog1-2* and *mft2*. (b) Wild type (Col-0) and the clock mutants, *toc1-1-1, lhy20 cca1-1,* *lhy20 cca1-1 toc1-2* and *prr5-11 prr7-11 prr9-10*. Data from Fig. 3 and Fig. S1 and S2 are replotted against thermal time (sum of temperature above 0°C) for secondary dormancy induction at 20, 25 and 30°C. The *dog1-2* mutant fits a linear regression (R=0.928) y=88.644+(-0.125)x. While the wold type Col-0 and all other mutants fit an exponential decay (3 parameter) regression. Col-0; (R2= 0.972) y=0.800+83.637 *exp(-0.018*x); *toc1-101*; (R2=0.881) y=-15.016+112.0598*exp(-0.0078*x); *lhy20 cca1-1*; (R2=0.916) y=-4.43+89.532*exp(-0.008*x); *lhy20 cca1-1 toc1-2*; (R2=0.897) y=-13.826+99.708*exp(-0.008*x); *prr5-11 prr7-11 prr9-10*; (R2=0.860) y=-20.394+142.272*exp(-0.005*x); *mft2*; (R2= 0.811) y=19.23+69.275*exp(-0.008*x). All regression were significant at (p<0.05).
