# Supplementary material for: A laboratory simulation of Arabidopsis seed dormancy cycling provides new insight into its regulation by clock genes and the dormancy‐related genes DOG1, MFT, CIPK23 and PHYA
Source: Plant Cell Environ. 2017 May 16;40(8):1474–86. doi: 10.1111/pce.12940 (PMC5518234; doi:10.1111/pce.12940)
Supplement: Supplementary file 10 — Figure S7. ABA sensitivity of Col‐0 and Ler wild types and CCA1 and LHY overexpressing lines. [file PCE-40-1474-s007.docx]

**Figure S7. ABA sensitivity of Col-0 and Ler wild types and *CCA1* and *LHY* overexpressing lines.** Following three days at 5°C/dark on water seeds were transferred to ABA (10 – 250 nM) in buffer at pH 5.0 and incubated at 25°C/light. (a) Germination is shown at each concentration after 14 days. (b) The time to 50% germination in hours (h) of data in (a). (c) Cumulative germination in the presence of 50 nM ABA. Data are mean ± SE (n = 3). Absence of error bars indicates SE is smaller than the symbol.
