# Supplementary material for: A laboratory simulation of Arabidopsis seed dormancy cycling provides new insight into its regulation by clock genes and the dormancy‐related genes DOG1, MFT, CIPK23 and PHYA
Source: Plant Cell Environ. 2017 May 16;40(8):1474–86. doi: 10.1111/pce.12940 (PMC5518234; doi:10.1111/pce.12940)
Supplement: Supplementary file 11 — Figure S8. Dark germination of Col‐0, and the dormancy mutants dog1–2 and mft2. [file PCE-40-1474-s008.docx]

**Figure S8. Dark germination of Col-0, and the dormancy mutants’ *dog1-2* and *mft2*.** Following cold treatment at 5°C/dark at -1.0 MPa, seeds were transferred to 25°C and 30°C in the dark and germination measured at the point of transfer to 25°C/light. (a) *dog1-2* and *mft2* at 25°C and 30°C. (b) Col-0 at 25°C and 30°C. Data are mean ± SE (n = 3). Absence of error bars indicates SE is smaller than the symbol.
