# Supplementary material for: A laboratory simulation of Arabidopsis seed dormancy cycling provides new insight into its regulation by clock genes and the dormancy‐related genes DOG1, MFT, CIPK23 and PHYA
Source: Plant Cell Environ. 2017 May 16;40(8):1474–86. doi: 10.1111/pce.12940 (PMC5518234; doi:10.1111/pce.12940)
Supplement: Supplementary file 12 — Figure S9. Dark germination of Col‐0, clock mutants and CCA1 and LHY overexpressing lines. [file PCE-40-1474-s009.docx]

**Figure S9. Dark germination of Col-0, and clock mutants and *CCA1* and *LHY* overexpressing lines.** Dark germination was measured on transfer to 20°C and 25°C in the dark. (a) Clock mutants at 20°C/dark. (b) Over expressing lines at 20°C/dark. (c) Clock mutants at 25°C/dark. (d) Over expressing lines at 25°C/dark. Data are mean ± SE (n = 3). Absence of error bars indicates SE is smaller than the symbol.
