# Supplementary material for: A laboratory simulation of Arabidopsis seed dormancy cycling provides new insight into its regulation by clock genes and the dormancy‐related genes DOG1, MFT, CIPK23 and PHYA
Source: Plant Cell Environ. 2017 May 16;40(8):1474–86. doi: 10.1111/pce.12940 (PMC5518234; doi:10.1111/pce.12940)
Supplement: Supplementary file 13 — Figure S10. DOG1 transcript level in Col‐0 seedlings entrained to a light/dark cycle. [file PCE-40-1474-s010.docx]

**Figure S10. *DOG1* transcript level in Col-0 seedlings entrained to a light/dark cycle.** Seedlings were sampled on the second and third day of constant light (60 µM m^-2^s^-1^) after growth for eight days in a 12h light/dark cycle at 22°C (n=1). Data from Edwards and Millar 2006 (<http://bar.utoronto.ca/efp/cgi-bin/efpWeb.cgi?dataSource=Light_Series>).
